# Renal cell carcinoma metastasizing to solitary fibrous tumor of the pleura: a case report

**DOI:** 10.1186/1752-1947-5-248

**Published:** 2011-06-29

**Authors:** Christopher Kragel, Shi Wei

**Affiliations:** 1 Department of Pathology, University of Alabama at Birmingham, Birmingham, AL 35249-7331, USA

## Abstract

**Introduction:**

A tumor metastasizing to another malignancy is an uncommon phenomenon. Since it was first described in 1902, there have been fewer than 200 cases reported in the literature, with lung cancer metastasizing to renal cell carcinoma being the most frequently described pattern. Here we report a case of a solitary fibrous tumor of the lung acting as the recipient for a renal cell carcinoma. To our knowledge, this is the first reported case of such a combination and the second case involving a solitary fibrous tumor.

**Case presentation:**

A 58-year-old Caucasian man who developed a persistent dry cough presented to our hospital. Imaging studies revealed a large pleural-based mass in the left lung. A biopsy of the mass showed a spindle-cell lesion consistent with a solitary fibrous tumor. The patient underwent surgical excision of the 13 cm mass. The pathological examination confirmed the diagnosis of a solitary fibrous tumor but also demonstrated discrete foci of metastatic renal cell carcinoma. Until that point, a primary renal cell carcinoma tissue diagnosis had not been made and the initial radiological work-up was inconclusive.

**Conclusion:**

Awareness of the unusual phenomenon of tumor-to-tumor metastasis is important for practicing surgical pathologists, particularly in the evaluation of a mass lesion showing bimodal histology. This case also highlights the importance of careful examination of surgical specimens, as minute and unusual findings can direct patient care.

## Introduction

The coexistence of two primary neoplasms in one patient is not uncommon, and these tumors may even arise at the same anatomic site ("collision tumor"). However, tumor-to-tumor metastasis is an extremely rare but interesting phenomenon. Since first described by Berent in 1902 [1], fewer than 200 cases have been reported in the English-language literature. The most frequent donor tumor site is the lung, while renal cell carcinoma is by far the most common recipient [2,3]. This combination constitutes approximately one-third of all reported cases. However, renal cell carcinoma acting as a donor tumor is extraordinarily rare, with only nine cases reported to date [4-12]. Interestingly, meningiomas are the most frequent recipients of donor renal cell carcinoma, followed by papillary carcinoma of the thyroid. Here we report the first case of a solitary fibrous tumor of the lung acting as the recipient of a donor renal cell carcinoma.

## Case presentation

**Figure 1:**
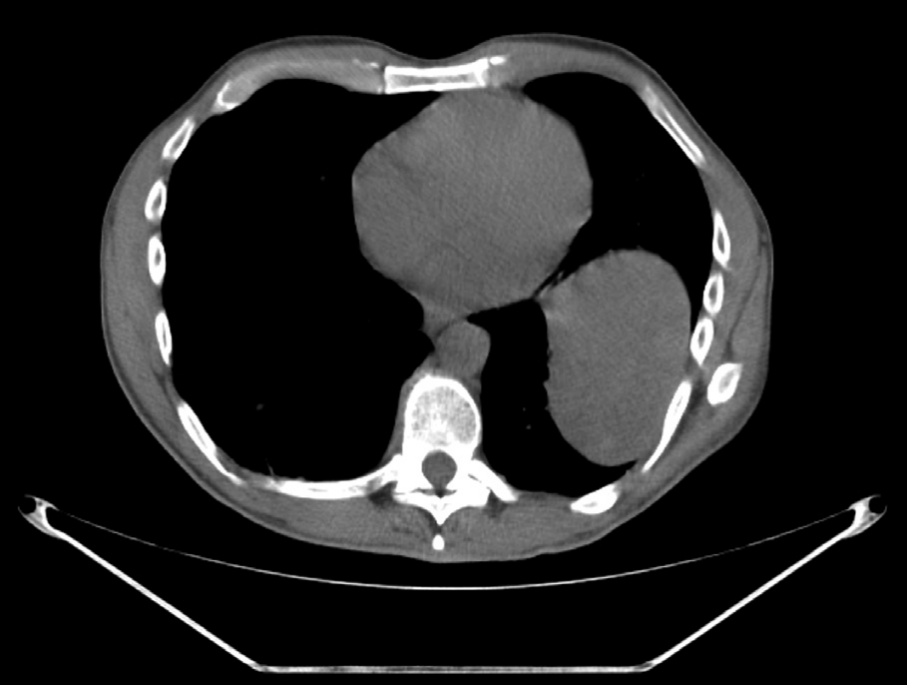
**Computed tomographic scan revealing a large pleural-based mass in the left hemithorax**.

**Figure 2:**
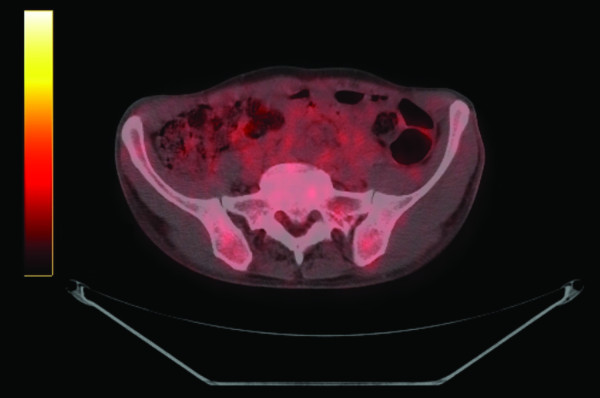
**Positron emission tomographic scan.** The left pleural mass showed only diffuse low-level fluorodeoxyglucose (^18^F-FDG) uptake of the mass. However, there was a focus of moderate ^18^FFDG activity in the superior aspect of the lesion, which was worrisome for malignancy.

A 58-year-old Caucasian man who developed a persistent dry cough and hemoptysis presented to our hospital. Computed tomography (CT) revealed a large, pleural-based mass in the left lung (Figure 1). A needle biopsy showed a spindle-cell neoplasm which was immunoreactive with CD34 and thus mostly consistent with a solitary fibrous tumor. The patient underwent further radiological work-up. Whole-body positron emission tomography (PET) showed diffuse, low-level fluorodeoxyglucose (^18^F-FDG) uptake of the large, biopsy-proven, solitary fibrous tumor of pleura in the left hemithorax. However, there was a focus of moderate ^18^F-FDG uptake in the superior aspect of the lesion, which was worrisome for malignancy (Figure 2). In addition, subcarinal necrotic lymphadenopathy was noted, which raised suspicions of metastasis. Multiple non-^18^F-FDG-avid large bilateral renal cysts were evident. The evaluation of other organ systems was unrevealing.

**Figure 3:**
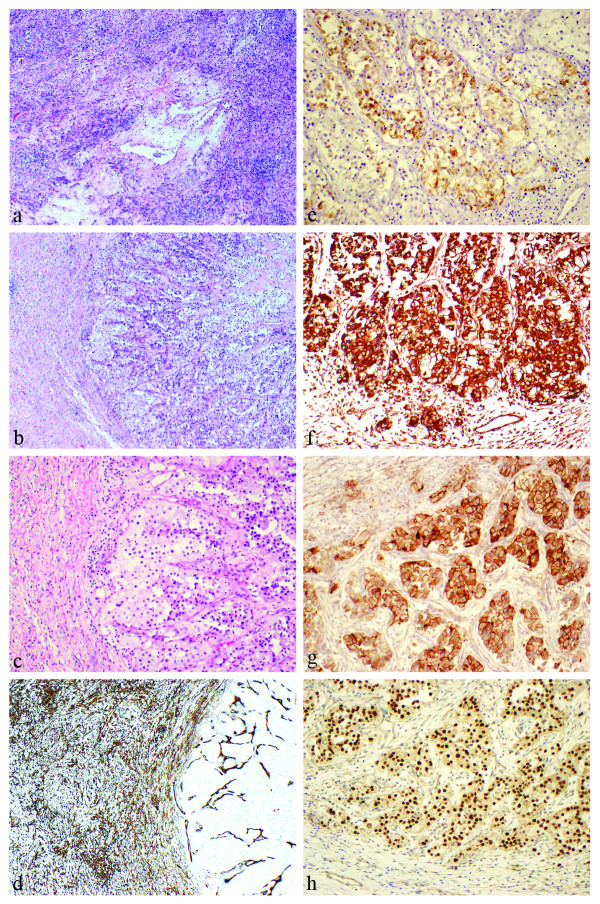
**Histologic and immunophenotypic characteristics of the tumor. (a)** Sections of pleural-based solitary fibrous tumor showing a cellular spindle-cell neoplasm with a patternless architecture and "staghorn" vessels. **(b and c) **A nodular collection of epithelioid clear cells was incidentally found within the tumor. **(d) **These cells are negative for CD34 (in contrast to the solitary fibrous tumor on the left), but immunoreactive with **(e)** broad-spectrum cytokeratin, **(f) **vimentin, **(g) **CD10 and **(h) **paired box gene 2 (PAX2).

The patient underwent surgical excision of the tumor, including left thoracotomy, partial pleurectomy, wedge resection of left upper and lower lobes and thoracic lymphadenectomy. Grossly, the tumor was homogeneously tannish-white and solid, measuring 13.0 cm × 9.0 cm × 6.0 cm. Microscopic examination revealed a cellular mesenchymal neoplasm composed of bland spindled cells with a patternless architecture. The lesion possessed "staghorn" vessels and a hyalinized stroma, especially in the peri-vascular regions (Figure 3a). The lesional cells were strongly immunoreactive with CD34 (Figure 3d). Thus, the features were characteristic of a solitary fibrous tumor.

Within the solitary fibrous tumor, there were two microscopic foci of nested epithelioid cells with clear cell features in the background of a delicate vascular network (Figures 3b and 3c). To further explore the nature of these cells, a battery of immunohistochemical staining was performed. The cells of interest were positive for broad-spectrum cytokeratin (Figure 3e) and vimentin (Figure 3f) and were also immunoreactive with CD10 (Figure 3g) and paired box gene 2 (PAX2) (Figure 3h). Thus, these cells most likely represented metastatic clear cell renal cell carcinoma. One lymph node showed necrotizing granulomata, but all thoracic nodes were negative for malignancy.

Post-operatively, a multidisciplinary team weighed the treatment options. However, in the coming months, further imaging analysis revealed additional metastases to the liver, spine and brain. The patient underwent chemotherapy, spinal radiation therapy and gamma knife radiosurgery for brain metastasis. With metastatic disease causing increased morbidity and no further treatment options available, the patient was placed in hospice care and died within six months of the initial diagnosis.

## Discussion

In 1968, Campbell *et al*. [13] reviewed previously reported cases and asserted the criteria for tumor-to-tumor metastasis as follows: (1) the existence of more than one primary tumor, (2) the recipient tumor is a true neoplasm, (3) the donor tumor is a true metastasis with established growth in the host tumor that is not the result of contiguous growth ("collision tumor") or embolization of tumor cells and (4) tumors that have metastasized to the lymphatic system, where a lymphoreticular malignant tumor already exists, are excluded. Thus, our present case meets these criteria.

Virtually any tumor may become a potential recipient of a donor metastatic tumor, but renal cell carcinoma is by far the most common one [2,3]. This is likely because kidneys receive significant blood flow and renal cell carcinoma is typically vascularly rich and thus easily harbors circulating tumor emboli [2,3]. It has also been suggested that the high glycogen and lipid content of carcinoma cells may serve as a suitable environment for metastatic deposits [14] and thus may reflect the "seed and soil" hypothesis of cancer metastasis [15]. A solitary fibrous tumor is extraordinary rare as a recipient tumor, and our present case report represents only the second reported such case. The tumor typically has alternating hypercellular and hypocellular areas and characteristic branching, staghorn vessels which may captivate blood-borne metastases, as in the case of renal cell carcinomas.

As a donor tumor, however, renal cell carcinoma is extremely uncommon, with only nine cases reported in the literature to date. All four patients with known follow-up died of the disease [4,6-8], which is compatible with the significantly unfavorable prognosis of other stage IV renal cell carcinomas. Interestingly, tumors of central nervous system [4,5,7,9,12] and thyroid carcinomas [8,10,11] represent frequent recipient tumors for donor renal cell carcinomas, suggesting that these organs and tumoral tissues may provide a fertile substrate or are some way predisposed targets for secondary growth of renal cell carcinoma.

The diagnosis of renal cell carcinoma metastasizing to solitary fibrous tumor is paramount in this case as the metastasis was the first confirmation of renal cell carcinoma in this patient. Retrospectively, the renal cysts identified in the initial radiological work-up may represent cystic renal cell carcinoma. This case exemplifies the importance of careful scrutiny of the pathologic specimens because rare or unusual pathologic findings may be of utmost clinical importance. In addition, our present case report also emphasizes the need for adequate sampling (that is, one section per centimeter of tumor mass), as only one of the 14 sections of the tumor possessed small metastatic foci.

## Conclusions

Awareness of the unusual phenomenon of tumor-to-tumor metastasis is important for practicing surgical pathologists, particularly in the evaluation of a mass lesion showing bimodal histology. This case also highlights the importance of careful examination of surgical specimens, as minute and unusual findings can direct patient care. Moreover, the relative frequency of specific neoplasms involved in tumor-to-tumor metastasis may shed light on the pathogenesis of tumor metastasis.

## Consent

Written informed consent was obtained from the patient for publication of this case report and any accompanying images. A copy of the written consent is available for review by the Editor-in-Chief of this journal.

## Competing interests

The authors declare that they have no competing interests.

## Authors' contributions

CK and SW were the responsible pathology resident and attending pathologist, respectively, of this patient, and both authors were major contributors to the manuscript. Both authors read and approved the final manuscript.

## References

[B1] Berent W Seltene metastasenbildung Zentralbl Allg Pathol 1902 13 5

[B2] Petraki C Vaslamatzis M Argyrakos T Petraki K Strataki M Alexopoulos C Sotsiou F Tumor to tumor metastasis: report of two cases and review of the literature Int J Surg Pathol 2003 11 127 135 10.1177/106689690301100214 12754635

[B3] Sella A Ro JY Renal cell cancer: best recipient of tumor-to-tumor metastasis Urology 1987 30 35 38 10.1016/0090-4295(87)90568-1 3603907

[B4] Osterberg DH Metastases of carcinoma to meningioma J Neurosurg 1957 14 337 343 10.3171/jns.1957.14.3.0337 13429401

[B5] Breadmore R House R Gonzales M Metastasis of renal cell carcinoma to a meningioma Australas Radiol 1994 38 144 3 10.1111/j.1440-1673.1994.tb00157.x 8024511

[B6] Ozenc A Ruacan S Baykal A Renal cell carcinoma and ipsilateral pheochromocytoma with neoplasm-to-neoplasm metastasis J Urol 1997 157 1831 1832 10.1016/S0022-5347(01)64871-7 9112533

[B7] Franke FE Altmannsberger M Schachenmayr W Metastasis of renal carcinoma colliding with glioblastoma. Carcinoma to glioma: an event only rarely detected Acta Neuropathol 1990 80 448 452 10.1007/BF00307701 2173331

[B8] Baloch ZW LiVolsi VA Tumor-to-tumor metastasis to follicular variant of papillary carcinoma of thyroid Arch Pathol Lab Med 1999 123 703 706 10420227 10.5858/1999-123-0703-TTTMTF

[B9] Han HS Kim EY Han JY Kim YB Hwang TS Chu YC Metastatic renal cell carcinoma in a meningioma: a case report J Korean Med Sci 2000 15 593 597 11069000 10.3346/jkms.2000.15.5.593 PMC3054676

[B10] Ryska A Cáp J Tumor-to-tumor metastasis of renal cell carcinoma into oncocytic carcinoma of the thyroid: report of a case and review of the literature Pathol Res Pract 2003 199 101 106 12747472 10.1078/0344-0338-00361

[B11] Bohn OL De las Casas LE Leon ME Tumor-to-tumor metastasis. Renal cell carcinoma metastatic to papillary carcinoma of thyroid: report of a case and review of the literature Head Neck Pathol 2009 3 327 330 10.1007/s12105-009-0147-9 20596854 PMC2811566

[B12] Kimiwada T Motohashi O Kumabe T Watanabe M Tominaga T Lipomatous meningioma of the brain harboring metastatic renal-cell carcinoma: a case report Brain Tumor Pathol 2004 21 47 52 10.1007/BF02482177 15696969

[B13] Campbell LV Jr Gilbert E Chamberlain CR Jr Watne AL Metastases of cancer to cancer Cancer 1968 22 635 643 10.1002/1097-0142(196809)22:3<635::AID-CNCR2820220320>3.0.CO;2-O 5673241

[B14] Ottosson L Berge T Metastasis from carcinoma to carcinoma Acta Pathol Microbiol Scand 1968 73 481 488 5697606 10.1111/j.1699-0463.1968.tb03207.x

[B15] Paget S The distribution of secondary growths in cancer of the breast Lancet 1889 133 571 573 10.1016/S0140-6736(00)49915-0 2673568

